# Uricase-deficient rats with similarly stable serum uric acid to human’s are sensitive model animals for studying hyperuricemia

**DOI:** 10.1371/journal.pone.0264696

**Published:** 2022-03-03

**Authors:** Yinfang Gao, Yun Yu, Wan Qin, Nan Fan, Yalin Qi, Huan Chen, Weigang Duan

**Affiliations:** 1 The Department of Pharmacology, School of Basic Medicine, Kunming Medical University, Kunming, Yunnan Province, China; 2 The Department of Pathology, School of Basic Medicine, Yunnan University of Traditional Chinese Medicine, Kunming, Yunnan Province, China; University Medical Center Utrecht, NETHERLANDS

## Abstract

The aim of this study was to provide a sensitive model animal for studying hyperuricemia. Male uricase-deficient rats, named Kunming-DY rats, were raised for 130 days, or orally administered with purines and other chemicals. Serum uric acid (SUA) in the animals was assayed, and the UA level in their organs and their 24-h excretion was determined. Genes in the jejunum, ileum, kidney and liver related to UA synthesis and transportation were detected by quantitative RNA sequencing. Uricase-deficient rats have a high level of SUA and are sensitive to xanthine, adenosine, inosine, allopurinol, and alcohol. Besides, the high level of SUA in male uricase-deficient rats was stable, much higher than that in wild-type rats but similar to that in men. The distribution pattern of UA in uricase-deficient rats’ organs was different from that in wild-type rats. The kidney, liver, and small intestine were the top three organs where UA distributed, but the UA in the small intestine, colon, lung, thymus, and brain was less affected by uricase deficiency, indicating that these organs are constitutive distribution organs in UA. The 24-h UA excreted by a uricase-deficient rat was about five times higher than that excreted by a wild-type rat. However, the 24-h UA excreted through feces was not significantly changed. Both the urine volume and UA in uricase-deficient rats significantly increased, and more than 90% of UA was excreted via urine. The expression of xanthine dehydrogenase was not upregulated. Some genes of transporter associated with uric acid excretion in the kidney were significantly regulated, though not sufficient to explain the increase in SUA. In conclusion, male uricase-deficient rats’ UA metabolism is similar to that of men. The elevation of SUA in uricase-deficient rats is caused by uricase deficiency, and uricase-deficient rats are a sensitive model for studying hyperuricemia.

## Introduction

Gout and other hyperuricemia-associated disorders are common threats to human health, and cause a heavy economic burden on modern society. Hyperuricemia occurs mostly in men, and it refers to a serum uric acid (SUA) level above 70 μg/ml for men and 60 μg/ml for women [1, 2]. This disorder is believed to be the direct foundation of gout because the relationship between hyperuricemia and gout is not only proved by numerous epidemiological studies, but also by clinical practice[1, 3]. Individuals with SUA levels below 360 μmol/L (about 60 μg/ml) have almost no risk of developing gout [3], although the relationship between hyperuricemia and the disease is still not fully clear [4]. Further clinical evidence has shown that hyperuricemia is also a risk factor for hypertension [5, 6], diabetes mellitus [6, 7], and other disorders [8].

Uric acid is an end product of purines in humans. In rats and mice, uric acid can be further transformed to 5-hydroxyisourate [9] and then to allantoin, two chemicals more soluble than uric acid. It is believed that more than two-thirds of uric acid is synthesized from endogenous purines, which are mainly associated with cell turnover [10]. Only less than one-third is transformed from exogenous purines; that is, from those obtained food [8]. The level of SUA in animals with uricase is not sensitive to endogenous and exogenous purines, and these animals seldom naturally develop hyperuricemia, gout, or associated disorders even if they are fed with a high-purine diet. Because of the deficiency of uricase, humans are sensitive to endogenous and exogenous purines; a fast cell turnover [11] or an intake of purine-rich food [12] can easily increase SUA to a high level, and even ignite a gout attack.

One of the best choices for studying the mechanisms of hyperuricemia and other associated disorders is to use animal models. The more closely the model mimics the disorder, the more likely the mechanism will be unveiled when studied. Considering their close genetic relationship and their relatively inexpensive price, rats and mice are the ideal experimental animals frequently used for basic medical research. Some hyperuricemia models have been established in rats or mice by oral purines, high-purine diets, uricase inhibitors, or/and uric acid excretion inhibitors [13]. However, the SUA level in normal animals is much lower than that in humans, and the SUA level of the “hyperuricemia” models can hardly meet the diagnostic criteria (70 μg/ml or 420 μmol/L for men) [13]. Actually, the baseline level of SUA in humans is high, and easily increases to a higher level to diagnose hyperuricemia. Therefore, different from the models mentioned above, clinical hyperuricemia can spontaneously occur, although it is usually tangled with some predisposing factors, such as alcohol and overeating [12].

Other hyperuricemia models have been established with uricase-deficient (Uox^-/-^) mice [14, 15]. However, the SUA level in the reported mice is too high to survive long periods. Specifically, the animals have significant renal injury caused by serious hyperuricemia (SUA above 100 μg/ml) [14, 15]. The Uox^-/-^ mice’s SUA level further increases with age, accompanied by obvious renal injury [16]. It is worth noting that the animals should be kept in a sterile environment [14], which results in a high economic cost of breeding. Because of their very high level of SUA, the animals are a naturally pathological model, and are not suitable for exploring the pathogenesis of hyperuricemia. Therefore, obviously, the models mentioned above poorly mimic human hyperuricemia.

To mimic human SUA levels, our team generated a new type of uricase-deficient animal based on Sprague-Dawley (SD) rats [17]. This animal was named the Kunming-DY rat. The SUA levels in the male animals are much higher than in the wild-type animals, but similar to those in men. It seems that Kunming-DY rats could be an alternative model animal to mimic human purine metabolism; they can be used as model animals not only to explore the mechanism of hyperuricemia and other associated disorders, but also to screen or evaluate drugs with possible SUA-lowering effects. The present study explored whether these rats can be used as an alternative model animal for the study of hyperuricemia, including the stability of the rats’ SUA and their sensitivities to purines, xanthine dehydrogenase inhibitor, and other factors.

## Materials and methods

### Materials

The parent wild-type SD rats were obtained from Chengdu Dossy Experimental Animals Co., Ltd, Chengdu, China [Certification No. SCXK (Chuan) 2008–24]. Uox^-/-^ (Kunming-DY) and wild-type 45-day-old rats were provided by our laboratory, as previously described [17].

Uric acid assay kits that use the phosphotungstic acid method (Lot: C012-1-1), creatinine assay kits that use the sarcosine oxidase method (C011-2-1), and urea assay kits that use the urease method (C013-2-1) were purchased from Nanjing Jiancheng Bioengineering Institute (Nanjing, China). A TRIzol Plus RNA Purification kit was purchased from Invitrogen (Carlsbad, CA, USA). Tris, tris acetate, xanthine, allopurinol, adenosine, and inosine of analytical purity were manufactured by Shanghai Yuanye Bio-Technology Co., Ltd. (Shanghai, China). Hydrochloric acid of analytical purity was manufactured by Yunnan Yanglin Development Zone Shandian Pharmaceutical Co. Ltd. (Kunming, China). Sodium bicarbonate and acetic acid of analytical purity were manufactured by The Third Tianjin Chemical Reagent Factory (Tianjing, China). Sodium acetate of analytical pure were manufactured by Tianjin Beichen Founder Reagent Factory (Tianjing, China). Sodium chloride and ethyl alcohol of analytical purity were manufactured by Tianjin Fengchuan Chemical Reagent Technology Co., Ltd. (Tianjing, China). Alkaline mineral water for drinking (pH 9.3) was purchased from a supermarket.

Ultrapure water was produced with a Milli Q water purification system manufactured by the EMD Millipore Group (Darmstadt, Germany). The NanoDrop ND-1000 spectrophotometer used for experiments was manufactured by PeqLab (Erlangen, Germany). We used a multiple microplate reader of K6600A manufactured by Beijing Kai’ao Technology Development Co., Ltd. (Beijing, China). Other instruments or reagents used in the present study were made in China, if not stated otherwise.

### Study design

**Fig 1:**
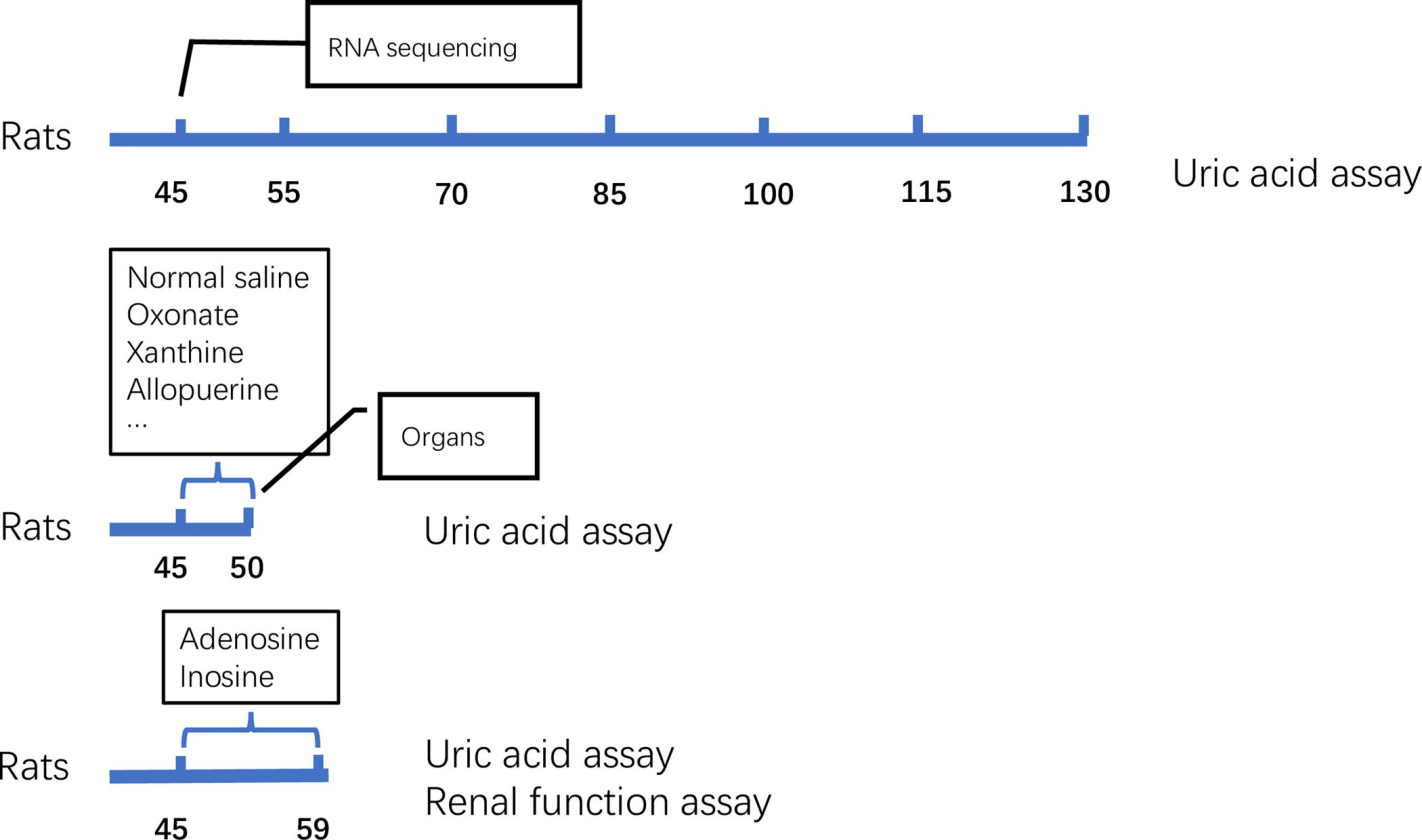
Study design.

First, SUA levels of rats at different ages were assayed to evaluate the stability of SUA in Uox^-/-^ rats. Second, rats were orally administered with oxonate, xanthine or allopurinol to test their sensitivity to factors that will affect SUA, and uric acid in their organs after administration were determined so as to evaluate organ contribution in uric acid metabolism. In addition, RNA in the concerned organs were quantitatively sequenced to investigate the expression of genes related to uric acid metabolism after uricase deletion (Fig 1). In the present study, wild-type rats of the same age were used as a control.

### Animal housing

Uox^-/-^ rats were provided by our laboratory [17]. Briefly, male Uox^-/-^ rats were mated with female Uox^-/-^ rats for three weeks to generate offspring. The offspring were breastfed to the age of 3 weeks by their mother; afterward, the mothers, the male offspring, and the female offspring were separated and put in three separate cages. When the male offspring were 45 days old, they were included in the study. The weight of the 45-day-old rats were usually 180–220 g.

Seven nests of male Uox^-/-^ rats and three nests of male wild-type rats were enriched in the present study. The rats were maintained at 22°C, with a humidity of 45−55% under natural light and a free approach to food and water. All animal experiments were approved by the Animal Care and Use Committee of Kunming Medical University (Approved No. KMMU-2020196), and were performed under the Guidelines for Ethical Review of Laboratory Animal Welfare of China.

All rats after the experiment were intraperitoneally anesthetized with urethane (1.0 g/kg). When they were under deep anesthesia, their necks were dislocated for euthanasia. The rat bodies were collected in yellow plastic bags and kept in a refrigerator at −20°C until they were taken away by a green company for cremation.

### Samples collection

#### Serum sample collection

When the male animals were 45 days old or on schedule days, their serum samples were collected. To obtain their blood, the rats were kept in small cages, and blood samples of about 0.2 ml were drawn from the tail vein using a tiny needle without anesthetization at a local atmosphere of 28−32°C. Serum was harvested by spinning at 3,000g and 4°C for 5 min as soon as the blood coagulated.

#### Excretion sample collection

Rats were individually kept in metabolic cages to harvest their 24-h excreta. Urine and feces were collected in a cold insulation box on ice. The urine was stirred to a homogeneous state, and 1.2 ml was sampled. The original sample was quickly diluted 20 times with water to obtain the final sample for uric acid assay. The feces were weighed and mixed with three times of 100 mmol/L Tris solution in weight, and the mixture was stirred at a shaker at 100 rpm for 4 h to extract the uric acid in the feces. The mixture was spun at 5,000g and 4°C for 5 min, and the supernatant was collected as the sample for uric acid assay.

#### Organ sample collection

At the end of the animal experiment, the animals were sacrificed, and their abdominopelvic and thoracic cavities were opened. Their organs, including the kidney, liver, pancreas, stomach, spleen, jejunum, adrenal gland, ileum, lung, colon, thymus, heart, skeletal muscle, and blood were harvested. Animal organs were weighed, cut to pieces, and homogenized with 10 times of sodium bicarbonate solution (50 mmol/L) on ice. The homogenate was spun at 10,000g and 4°C for 10 min to obtain the supernatant.

The supernatant made from the fresh tissues and the samples for uric acid assay were kept at −20°C or determined as soon as possible in order to prevent the false elevation of uric acid levels by xanthine dehydrogenase [18].

## Uric acid assay

Uric acid in the samples mentioned above was assayed using the uric acid assay kits. The assay kits had a good quantification range of uric acid from 3.91 μg/ml to 125 μg/ml. If uric acid in the sample was above that range, the sample was diluted. The protocols of the uric acid assay kits are available at http://www.njjcbio.com/uploadfile/product/big/20190612093216738.pdf.

### SUA in rats affected by oral adenosine, inosine and other chemicals

Rats at the age of 45 days were administered with xanthine, potassium oxonate, or allopurinol for 5 days by intragastric gavage. Their blood samples were collected before administration and 2 h after the last administration. The uric acid in the serum samples was determined by the uric acid assay kits, as mentioned above.

The solutions of adenosine (2.2 g/L), inosine (2.2 g/L), tris acetate (0.5 and 1.5 g/L), tris hydrochloride (0.5 and 1.5 g/L), sodium bicarbonate (0.5 and 1.5 g/L), sodium acetate (0.5 and 1.5 g/L), sodium chloride (0.5 and 1.5 g/L), acetic acid (0.5 and 1.5 g/L), hydrochloride (0.5 g/L), and ethyl alcohol (4 g/L) were prepared. The above solutions and alkaline mineral water were freely drunk by the Uox^-/-^ rats. Their blood samples were collected before administration and at the end of the experiment. The uric acid level in the serum samples was also determined by the uric acid assay kits. Creatinine and urea in the serum were determined by the creatinine assay kits and the urea assay kits, and the protocols can be downloaded at http://www.njjcbio.com/uploadfile/product/big/20190612093006383.pdf (for the creatinine assay kits) and http://www.njjcbio.com/uploadfile/product/big/20190612093302775.pdf (for the urea assay kits).

### Abundance of genes expressed in organs

Animals at the age of 45 days were anesthetized with urethane (1.0 g/kg). When their abdomen was opened, their liver, kidney, jejunum (the first 3 cm of the small intestine), and ileum (the last 3 cm of the intestine) were harvested. The fresh organs were frozen with liquid nitrogen and ground to powders. The total RNA in the powders was extracted and purified by the TRIzol Plus RNA Purification Kit. The quantity and quality of RNA were measured by the NanoDrop ND-1000 spectrophotometer. RNA integrity was assessed by denaturing gel electrophoresis of RNA as previously described [19, 20].

Double-stranded cDNA (ds-cDNA) was reverse-transcribed from the total RNA using a SuperScript ds-cDNA synthesis kit (Invitrogen, Carlsbad, USA) in the presence of 100 pmol/L oligo dT primers. Solexa high-throughput sequencing technique was used to sequence the cDNA by Sangon Biotech Co. Ltd. (Shanghai, China). The raw data containing reads of 150 bases of nucleotides in fastq format were transformed to original sequences in fasta format by Seqkit software in a disc operation system (DOS) model [21]. The sequences matching 27 bp or more with the rat’s reference mRNA sequences (https://www.ncbi.nlm.nih.gov/) were screened out by TBtools software (v0.664445552). The expected value of fragments per kilobase of transcript sequence per million base pairs sequenced (FPKM) was used for normalization of the expression level [22, 23].

### Statistical analysis

Values were expressed as mean + standard deviation (SD) or Median + inter-quartile range (IQR). If the normal distribution of values was verified by the normality test (Shapiro-Wilk test), Student’s t-test (for two groups, two-tailed) or one-way analysis of variance (ANOVA) (for three or more groups) was performed to compare means between different groups. If there was a significant difference between three or more groups, post-hoc tests between every two groups were performed using the S-N-K method (equal variances assumed) or Tamhane’s T2 method (equal variances not assumed). Otherwise, nonparametric tests for two (Mann-Whitney U test) or several (Kruskal-Wallis H test) independent samples were performed. Statistical significance was accepted at P < 0.05.

## Results

### Uox^-/-^ rats have a high level of SUA and are sensitive to oral xanthine and allopurinol

**Fig 2:**
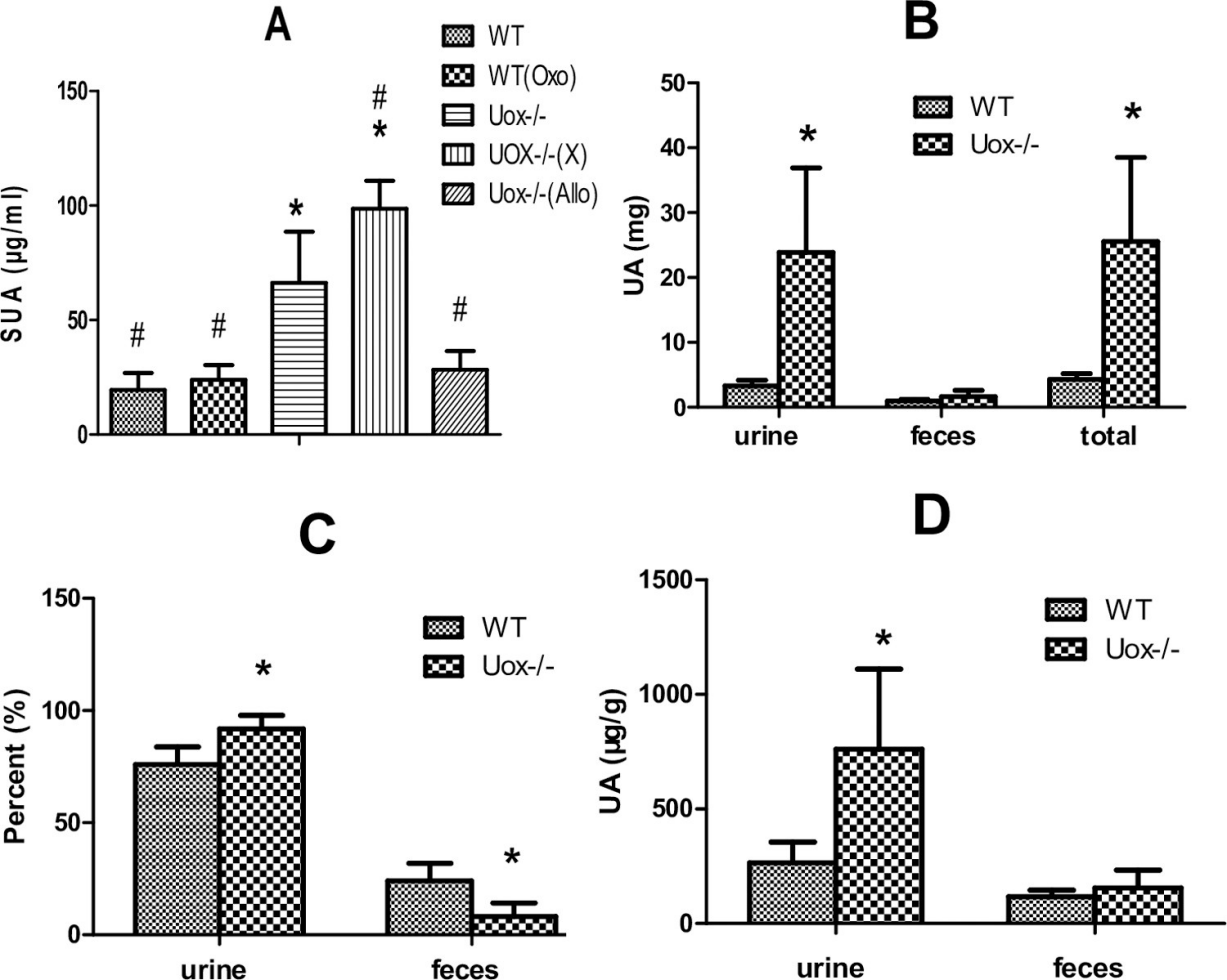
Uric acid in Uox^-/-^ rats’ serum and excrements (mean + SD). A, Serum uric acid (SUA) level in male wild-type rats or Uox^-/-^ rats (n = 6 for WT, WT(Oxo), Uox-/-(X) and Uox-/-(Allo) groups; or 9 for Uox-/- group), *, P<0.05 vs WT, ANOVA, #, P<0.05 vs Uox-/-, ANOVA. B, Amount of uric acid excreted from 24 h urine or feces (n = 6 for WT group, 9 for Uox-/- group), *, P<0.05 vs WT, Student’s t-test; C, The percent of uric excreted through urine and feces (n = 6 for WT group, 9 for Uox-/- group), *, P<0.05 vs WT, Student’s t-test; D, The concentration of uric acid in urine and feces, *, P<0.05 vs WT, Student’s t-test. WT, normal wild-type rats; WT(Oxo), wild-type rats treated with oral potassium oxonate (200 mg/kg, 5 days); Uox-/-, blank Uox^-/-^ rats; Uox-/-(X), Uox^-/-^ rats treated with oral xanthine (300 mg/kg, 5 days); Uox-/-(Allo), Uox^-/-^ rats treated with oral allopurinol (30 mg/kg, 5 days).

The SUA level in male wild-type rats was about 20 μg/ml, and was almost not affected by oral potassium oxonate, a uricase inhibitor, at 200 mg/kg for 5 days. Uricase deficiency caused a significant elevation of SUA level to about 65 μg/ml. The level of SUA in Uox^-/-^ rats was further elevated to about 100 μg/ml by oral xanthine at 300 mg/kg for 5 days, and lowered to about 30 μg/ml by oral allopurinol at 30 mg/kg for 5 days (Fig 2A).

The amount of total urine uric acid excreted by Uox^-/-^ rats in 24 h was significantly higher than that excreted by wild-type rats (Fig 2B). However, the amount of total fecal uric acid excreted by Uox^-/-^ rats in 24 h was almost not changed. The amount of total uric acid excreted by Uox^-/-^ rats in 24 h was about five times higher than that excreted by wild-type rats. Because of the increase of uric acid excretion, the percent between the amounts in uric acid excreted through urine and through feces was significantly changed (Fig 2C). In the wild-type rats, about 75% of uric acid was excreted through urine, while in the Uox^-/-^ rats, more than 90% was excreted through urine. Notably, the concentration of uric acid in in the urine of Uox^-/-^ rats was much higher than that in wild-type rats (Fig 2D).

### Uox^-/-^ rats are sensitive to oral adenosine and inosine

**Fig 3:**
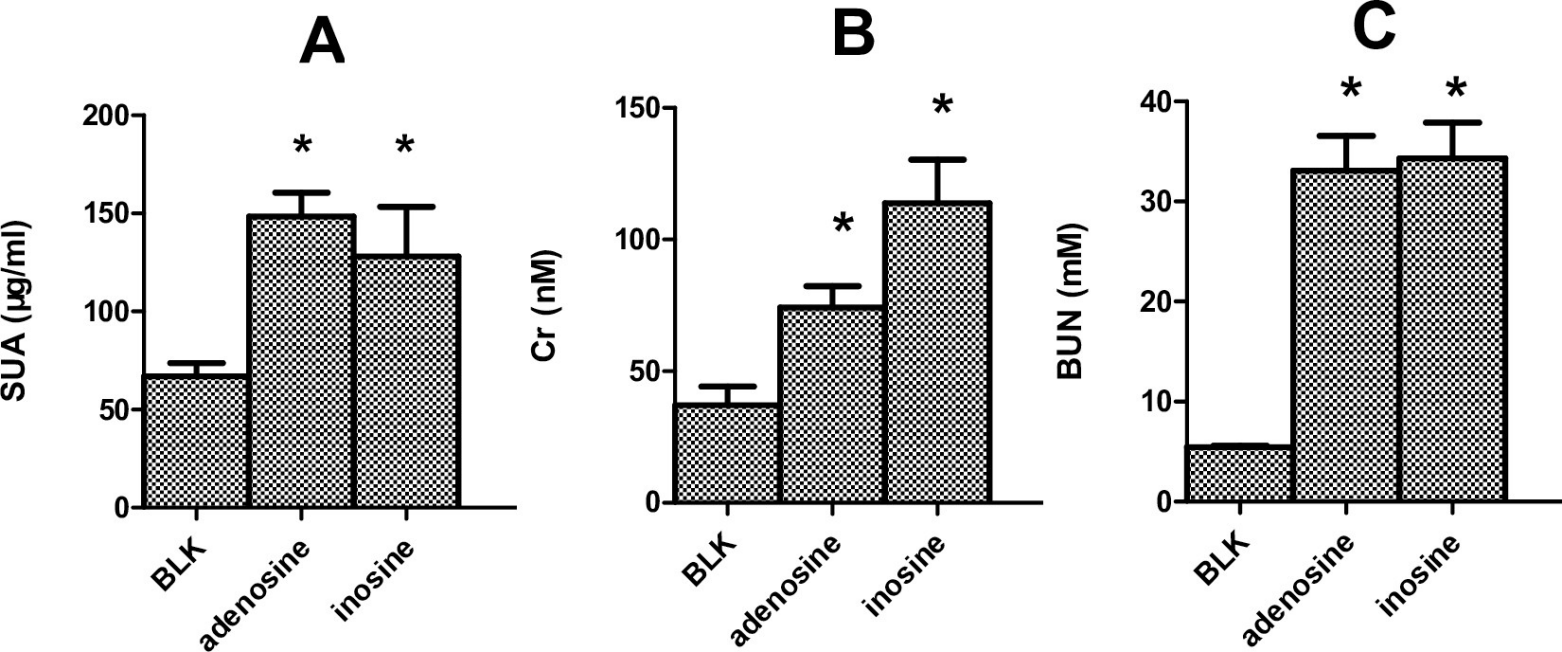
Uox^-/-^ rats treated with adenosine (2.2 g/L) or inosine (2.2 g/L) by free drinking for two weeks (mean + SD, n = 6). SUA (serum uric acid, A), Creatinine (Cr, B), and urea (BUN, C) affected by adenosine or inosine. Blk group, Uox^-/-^ rats without treatment. *, P < 0.05 vs Blk, ANOVA.

Uox^-/-^ rats’ SUA level (Fig 3A), together with creatinine (Fig 3B) and urea (Fig 3C), was significantly elevated by free drinking of adenosine or inosine (2.2 g/L) for 2 weeks. The increase in serum creatinine and urea suggested the rats’ renal injury. Different from rats treated with adenosine and inosine, the blank male Uox^-/-^ rats’ SUA level was similar to that of men, and their renal function was still in the normal range.

### Uox^-/-^ rats’ SUA level is affected by other factors

**Fig 4:**
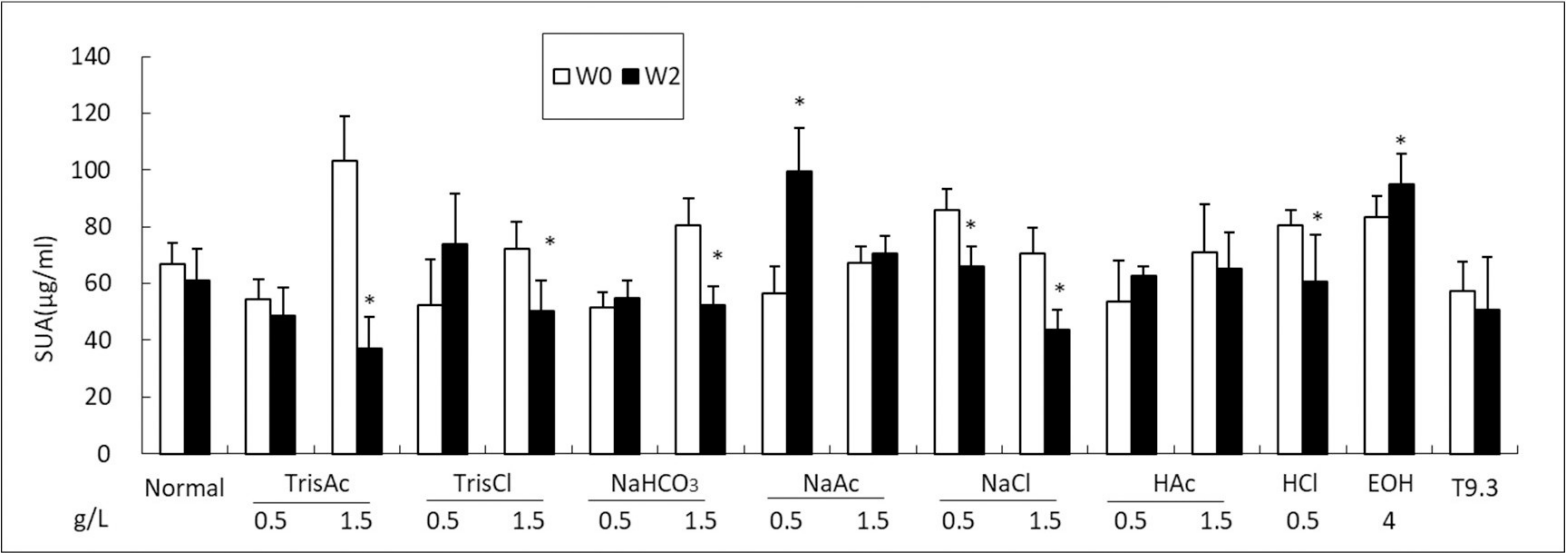
Uox-/- rats’ SUA affected by several factors by free drinking (mean + SD, n = 6). W0, before administration; W2, administered for 14 days. SUA, serum uric acid; Normal, Uox-/- rats administered with tap water; TrisAc, tris acetate; TrisCl, tris hydrochloride; NaHCO3, sodium bicarbonate; NaAc, sodium acetate; NaCl, sodium chloride; HAc, acetic acid; HCl, hydrochloride acid; EOH, alcohol; T9.3, alkaline mineral water with pH 9.3. *, P < 0.05 vs W0, Student t-test.

To evaluate the Uox^-/-^ rats’ reactivity to other factors, the rats were exposed to solutions of different concentrations by free drinking. After two weeks of free drinking, some factors significantly affected the Uox^-/-^ rats’ SUA level. As shown in Fig 4, tris acetate (TrisAc), tris hydrochloride (TrisCl), and sodium bicarbonate (NaHCO_3_) at 1.5 g/L lowered SUA by free drinking. Sodium acetate (NaAc) at a low concentration of 0.5 g/L was able to elevate SUA, but its ability ceased at a higher concentration. Unexpectedly, the intake of sodium chloride (NaCl) lowered SUA, although the intake is not believed to be helpful for cardiovascular system. Considering that the intake of both acetate and acetic acid did not lower SUA while hydrochloride and its acid lowered SUA, the intake of chloride ion could be helpful for uric acid lowering. As expected, the intake of alcohol significantly increased SUA in Uox^-/-^ rats. It should be noted that the alkaline mineral water (T9.3) did not lower SUA.

### SUA level is stably high in male Uox^-/-^ rats

**Fig 5:**
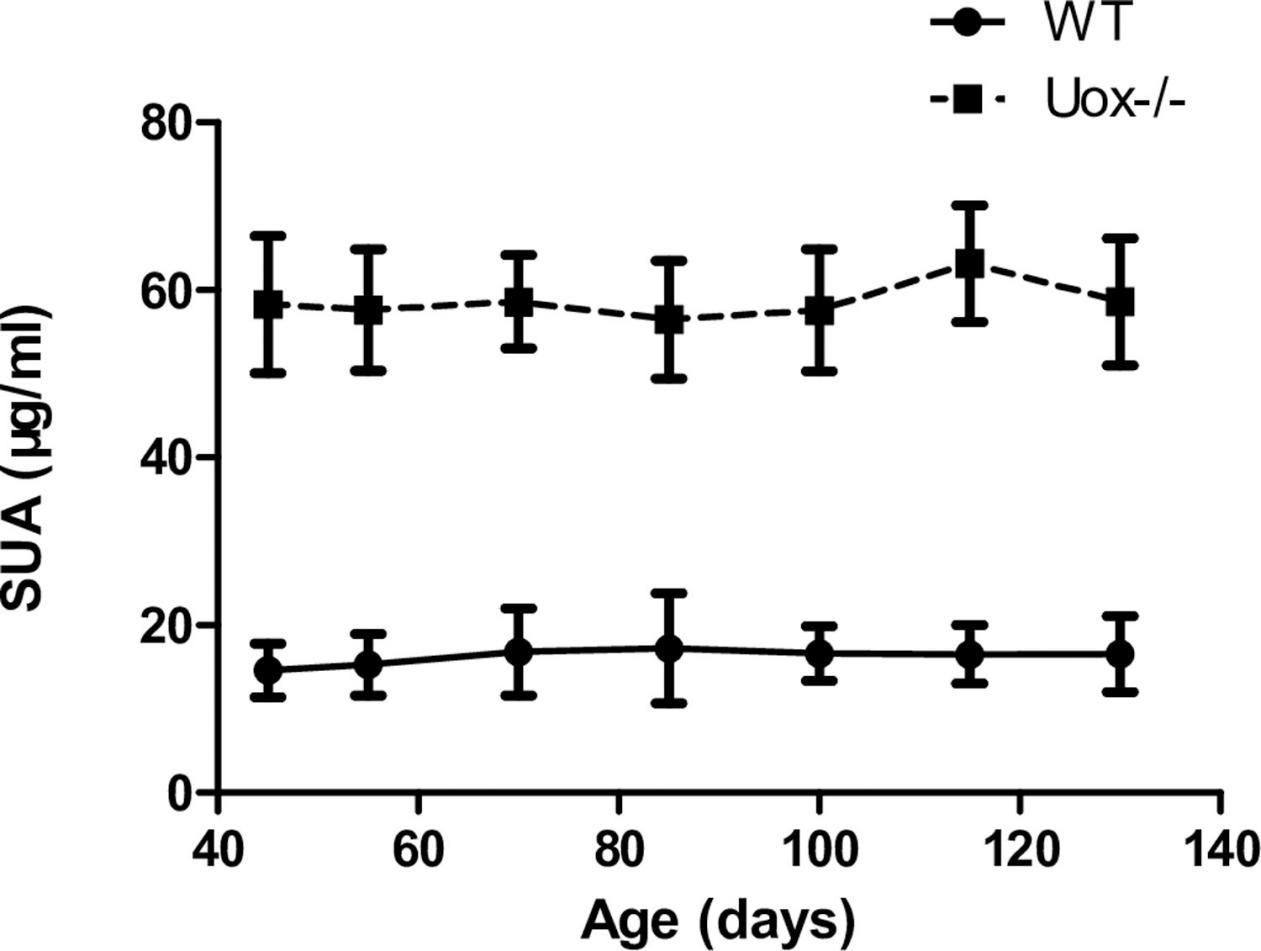
Chronic SUA in Uox^-/-^ rats (mean + SD, n = 8). SUA, serum uric acid; WT, normal wild-type rats; Uox-/-, blank Uox^-/-^ rats.

Considering that SUA level in 45-day-old Uox^-/-^ rats were significantly higher than in wild-type rats, it was necessary to evaluate whether SUA in Uox^-/-^ rats is stable enough for chronic hyperuricemia study. Fortunately, SUA in Uox^-/-^ rats was stably high between the age of 45 days and 130 days (Fig 5).

### Uric acid distribution in the organs of Uox^-/-^ rats

**Fig 6:**
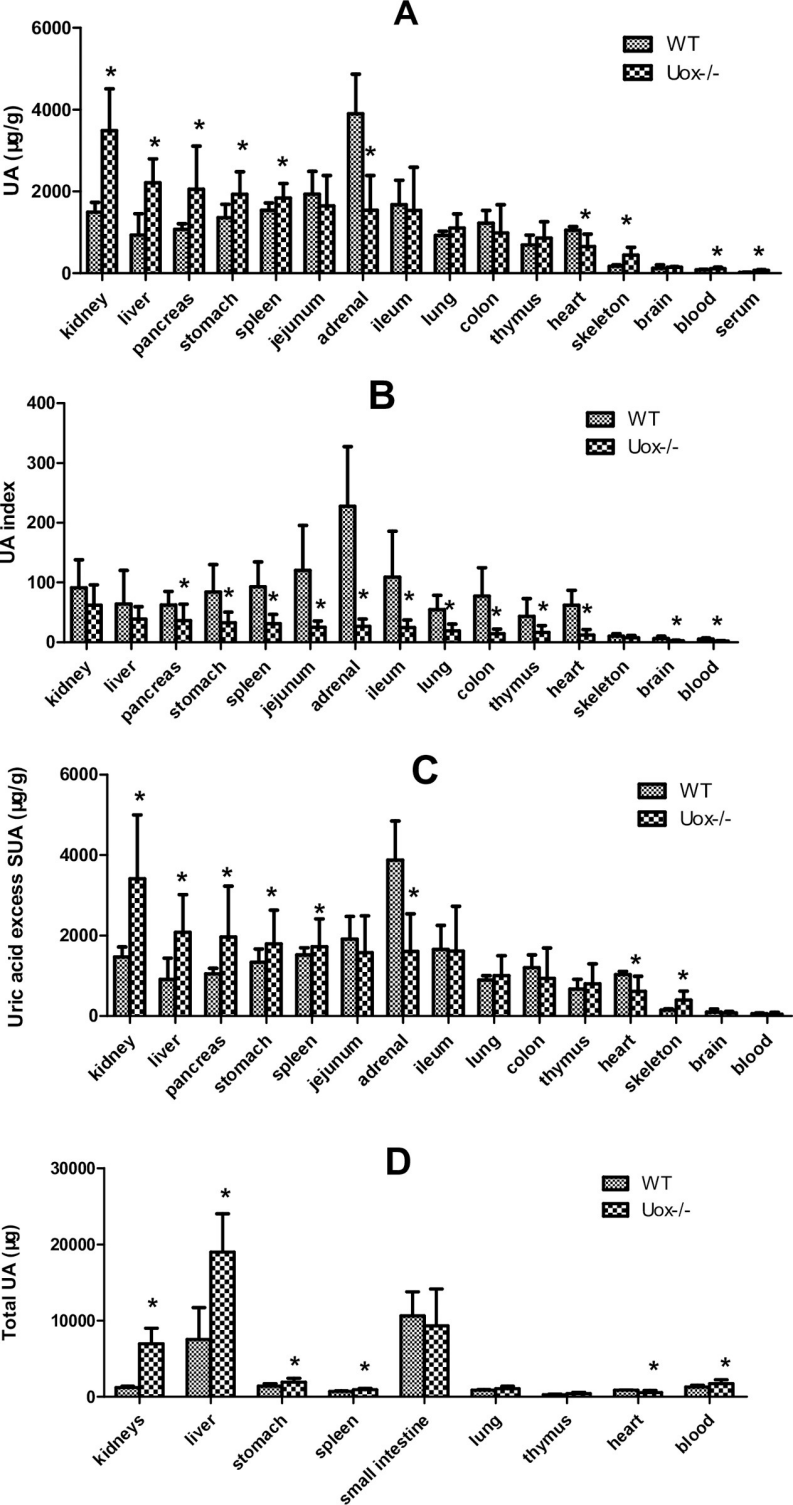
Uric acid distributed in blank Uox^-/-^ rat’s organs (mean + SD, n = 9 (WT) or 8 (Uox-/-)). A, Uric acid content (μg/g) in the organs. B, Uric acid index of the organ, which was calculated by dividing tissue uric acid content by its serum uric acid concentration. C, The part of uric acid content of the organ excessing their SUA, which was calculated by subtracting serum uric acid concentration from its tissue uric acid content. D, Total uric acid in the organ, which was calculated by multiplying tissue uric acid content by its organ weight. SUA, serum uric acid; WT, normal wild-type rats; Uox-/-, blank Uox^-/-^ rats. Skeleton muscle was the gluteus maximus. Serum uric acid in Fig 6A (the last column) was cited from Fig 2A. *, P<0.05 vs WT, Student’s t-test.

The pattern of uric acid distribution in Uox^-/-^ rats was different from that in wild-type rats (Fig 6A). Except in the adrenal glands and heart, the content of uric acid in Uox^-/-^ rats’ organs was overall higher than that in wild-type rats, though partly without significance. If the uric acid content in the organs was balanced with SUA to calculate the UA index, it was found that the UA indexes of the organs were overall lowered (Fig 6B).

The uric acid content in the organs was overall overwhelmingly higher than the SUA level. The part of the uric acid content of the organs excessing their SUA is shown in Fig 6C, which is very similar to Fig 6A. However, there was no significant difference in the excess uric acid in blood between wild-type rats and Uox^-/-^ rats. Since uricase activity can be detected in the wild-type rats’ serum [17], the increased uric acid content in Uox^-/-^ rats’ blood (Fig 6A) could be directly caused by the absent uricase in their blood.

Considering the mass of the organs, the total uric acid in an organ was calculated by multiplying the uric acid content by its weight [24]. According to the results shown in Fig 6D, the liver, small intestine, and kidneys were the top three organs in uric acid distribution. Interestingly, uric acid was constitutively distributed in the small intestine because the total uric acid in Uox^-/-^ rats’ small intestine was similar to that in wild-type rats. Furthermore, the lung, colon, thymus, and brain were the organs where uric acid was also constitutively distributed, because their uric acid content was also similar between in Uox^-/-^ and wild-type rats.

### Uric acid distribution in the organs of Uox^-/-^ rats treated with xanthine and allopurinol

**Fig 7:**
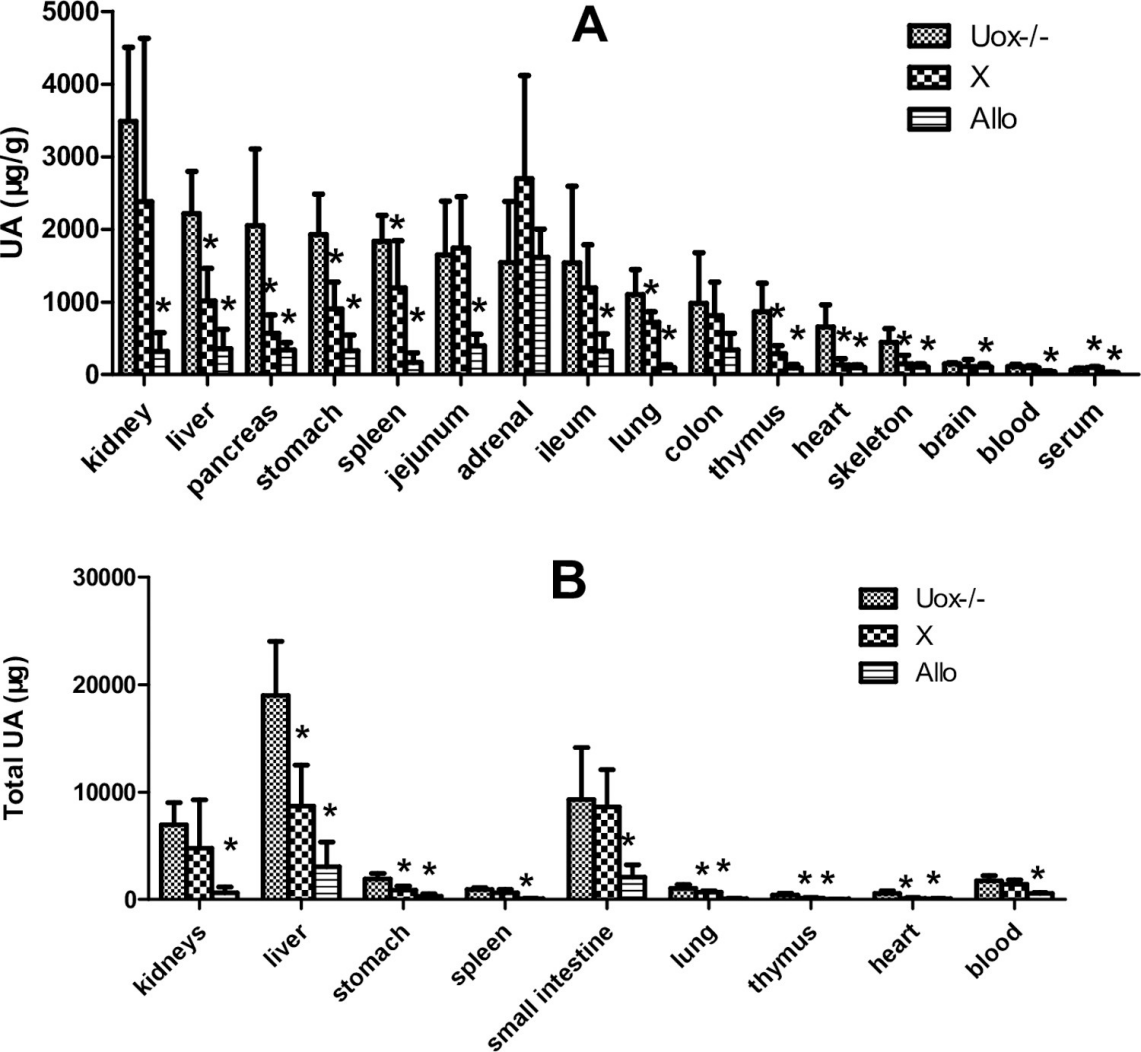
Uric acid distributed in Uox^-/-^ rat’s organs after orally administered with xanthine (300 mg/kg) or allopurinol (30 mg/kg) (Mean + SD, n = 8 (Uox-/- group) or 6 (X, and Allo groups). A, Uric acid content (μg/g) in the organs. B, Total uric acid in the organ, which was calculated by multiplying tissue uric acid content by its organ weight. SUA, serum uric acid; Uox-/-, blank Uox^-/-^ rats, cited from Fig 6; X, Uox^-/-^ rats treated with oral xanthine (300 mg/kg) for 5 days; Allo, Uox^-/-^ rats treated with oral allopurinol (30 mg/kg) for 5 days. Skeleton muscle was the gluteus maximus. X, xanthine; Allo, allopurinol; *, P<0.05 vs Uox-/-, ANOVA.

To observe whether the pattern of uric acid distribution was affected by exogenous drugs, Uox^-/-^ rats were treated with oral xanthine (300 mg/kg) or allopurinol (30 mg/kg) for 5 days. The results showed that the patterns of uric acid distribution in Uox^-/-^ rats treated with the two drugs were different from those in blank Uox^-/-^ rats (Fig 7A). Oral xanthine significantly increased SUA level in Uox^-/-^ rats (Fig 7A), but not in wild-type rats (Fig 2A). The uric acid content in most organs of Uox^-/-^ rats treated with xanthine was similar to, or slightly lower than, that in the blank Uox^-/-^ rats. However, uric acid distributed in the organs in Uox^-/-^ rats treated with allopurinol was much lower than that in the blank rats.

Considering the mass of the organs [24], the total uric acid in an organ was calculated by multiplying the uric acid content by its weight. As shown in Fig 7B, the liver, small intestine, and kidneys were still the top three organs in uric acid distribution. Interestingly, in Uox^-/-^ rats treated with xanthine, the total uric acid in the small intestine or kidney was similar to that in wild-type rats, but the total uric acid in the liver significantly decreased (Figs 6D and 7B). In Uox^-/-^ rats treated with oral allopurinol, the total amount of uric acid in organs was significantly lowered.

### Expression of genes associated with uric acid transportation, synthesis, and degradation in organs

Since the small intestine, liver, and kidney were shown to be the most important organs associated with uric acid distribution, whole genes expressed in these organs at RNA level were quantitively sequenced. Based on the current understanding [25, 26], the genes associated with uric acid transportation, synthesis, degradation, and purine recycle were sought out and listed in Table 1, and their expression abundance was also calculated.

**Table 1:** Genes associated with uric acid transportation, synthesis and degradation expressed at RNA level in Uox^-/-^ rats’ organs (FPKM, mean + SD, n = 3).

Gene Name	Duodenum		Ileum		Kidney^a^		Liver		Note
	WT	Uox^-/-^	WT	Uox^-/-^	WT	Uox^-/-^	WT	Uox^-/-^	
Abcc4	1.13+0.32	1.44+0.59	2.74+0.43	2.32+0.27	17.17+2.02	13.89+1.17	1.93+0.11	0.83+0.15*	Uric acid secretion
Abcg2	70.44+12.97	70.79+20.94	210.22+5.10	171.58+13.01*	71.19+10.03	79.90+8.91	10.53+1.48	6.56+2.36	
Lgals9	434.31+94.21	364.52+69.14	397.13+40.59	382.08+13.86	13.62+2.34	7.40+1.77*	103.81+19.48	51.38+6.33*	
Slc17a1	0+0	0+0	0+0	0.09+0.02	44.01+4.75	54.49+6.00	18.90+6.58	16.98+1.87	
Slc22a6	0+0	0.01+0.01	0.01+0.02	0.01+0.01	541.93+36.04	505.83+51.18	0.25+0.12	0.09+0.09	
Slc22a12	0+0	0+0	0.14+0.13	0.02+0.02	396.99+33.77	285.08+43.38*	0.02+0.04	0.01+0.01	Uric acid intake or reclamation
Slc22a13	0.14+0.06	0.11+0.08	0.22+0.20	0+0	11.67+3.25	3.42+0.05*	0+0	0.04+0.04	
Slc22a8	0+0	0.00+0.01	0.03+0.02	0.07+0.02	395.78+47.31	433.38+31.86	162.05+10.66	59.4+49.79*	
Slc2a6	1.70+0.30	2.15+0.15	1.61+0.26	2.25+0.11	1.03+0.06	0.88+0.09	0.65+0.06	0.41+0.05*	
Slc2a9	15.28+2.56	9.85+3.44	24.23+7.20	15.45+2.55	7.21+0.36	7.62+0.37	10.89+1.76	12.73+1.11	
Ada	1461.85+337.99	565.05+67.73*	230.33+78.40	129.10+39.59	9.59+2.00	8.01+1.62	3.15+0.61	1.81+0.25*	Uric acid synthesis
Xdh	261.88+15.60	215.45+33.92	89.74+14.30	84.33+2.78	35.45+3.80	37.95+3.84	32.08+1.73	25.38+3.00*	
Gda	87.59+28.73	202.35+13.64*	108.48+11.53	137.39+5.37*	5.743+1.53	8.23+1.25	13.06+2.05	14.58+2.38	
Aprt	262.86+6.75	176.53+36.51*	129.55+4.69	73.17+5.81*	153.43+9.38	90.08+17.55*	40.46+2.49	46.02+3.00	Purine recycle
Hprt1	14.24+3.90	10.61+0.61	16.25+2.53	11.15+1.06*	28.11+1.34	15.70+1.14*	21.11+3.37	21.45+4.50	
Pnp	283.31+16.90	259.42+43.41	118.31+8.25	103.47+2.96*	145.63+3.10	110.07+11.15*	197.43+18.42	128.14+22.89*	
Aox1	0.47+0.25	0.78+0.13	3.50+3.76	1.28+0.24	4.04+0.68	3.16+1.35	147.86+24.96	34.16+3.60*	Uric acid degradation
Uox ^b^	0.02+0.029	0+0	0.08+0.07	0+0	0.03+0.06	0.01+0.01	384.58+79.50	139.30+39.12*	

a, Cited from Yu et al. [17]

b, Expression of Uox in Uox^-/-^ rats was an invalid transcript, because its Exons 2–4 were deleted. WT, male wild-type rats of 45 days old; Uox^-/-^, male Uox^-/-^ rats.

*, P < 0.05 vs WT, Student t-test

As shown in Fig 6D, there was no significant difference in the total uric acid of the small intestine (including jejunum and ileum) between Uox^-/-^ rats and wild-type rats. Accordingly, the results in Table 1 showed that there were no transporters, including Slc17a1, Slc22a6, Slc22a12, Slc22a13, Slc22a8, Slc2a6, and Slc2a9, significantly regulated between them. Interestingly, a gene (Ada) associated with uric acid synthesis and some genes associated with purine recycle were significantly downregulated in the small intestine (including jejunum and ileum). Surprisingly, Gda, another gene associated with uric acid synthesis was upregulated in Uox^-/-^ rats (Table 1).

However, in the Uox^-/-^ rats’ liver, Abcc4 and Lgals9, transporters for uric acid secretion were downregulated, and the downregulation would facilitate uric acid accumulation in the liver. According to the results shown in Table 1, Slc22a8 and Slc2a6 in Uox^-/-^ rats’ liver were also significantly downregulated. Considering that both of them are passive transporters for uric acid intake and the content of uric acid in the liver is much higher than SUA, the downregulation would play a minor role in affecting the uric acid in the liver. The key gene for synthesizing uric acid, xanthine dehydrogenase (Xdh), was downregulated. The gene of Uox expressed in Uox^-/-^ rats was an invalid transcript of uricase because its Exons 2–4 were deleted [17]. Besides Uox, Aox1 [27] (aldehyde oxidase 1) is regarded as another enzyme that catalyzes uric acid, and the gene in Uox^-/-^ rats’ liver was downregulated.

In Uox^-/-^ rats’ kidney, Lgals9 (a transporter for uric acid secretion) was downregulated, and the transporters for uric acid intake (Slc22a12 and Slc22a8) were also downregulated. Their downregulation would likely have a complex impact on uric acid transportation in the kidneys. Interestingly, the genes associated with uric acid synthesis were downregulated, but the key gene encoding Xdh was not significantly affected.

## Discussion

### Male Uox^-/-^ rats’ SUA level is stable and similar to that of men

Hyperuricemia is a common disorder in humans. One of the best ways to understand its mechanism is to use model animals that can highly mimic purine metabolism in humans. Then, these animals can be used to replicate hyperuricemia by applying clinically relevant factors, and even to replicate associated diseases highly consistent with those in humans.

Male wild-type rats and mice were frequently used to establish models to study hyperuricemia and related disorders [13]. However, uricase expressed in the animals is a big obstacle that should be overcome. Uric acid can be eliminated by metabolism via uricase (and Aox1) and by excretion via urine and feces. However, according to the present study, uricase is responsible for about five-sixths of the total uric acid elimination in wild-type rats. After Uox deletion in rats, there was no obvious change in the expression of genes involved in uric acid synthesis and transport in the top three organs (the kidney, liver, and small intestine) with the largest distribution of uric acid. Therefore, it can be considered that the increase of blood uric acid in Uox^-/-^ rats is mainly caused by uricase deficiency. Obviously, uricase relieves the burden of uric acid on the kidneys.

Uricase inhibitors, such as oxonate, are often jointly used to raise SUA [13] so as to establish a hyperuricemia model. Because the uricase is an inducible enzyme [28], its activity cannot be fully inhibited by oxonate. In fact, few reports have shown that SUA level can stably raise to 50 μg/ml or more, which is still not enough for a real hyperuricemia model (SUA above 70 μg/ml). Moreover, the use of oxonate often places an additional extra burden on the liver and even causes other effects because the original effect of oxonate is to inhibit orotate phosphoribosyltransferase [29]; this further makes the model more complex.

The present study showed that, the uricase deficiency caused the rats’ SUA level to significantly increase to the levels occurring in humans with hyperuricemia. The level of SUA in the Uox^-/-^ rats was higher than that in the reported wild-type rats that were jointly administered with oxonate, and was maintained for at least 130 days. Considering that the uricase deficiency only causes a mild injury in rats [24], the results suggest that, like humans, the rats are apparently healthy, but vulnerable to renal injury factors.

Interestingly, according to the values calculated by the body surface area, the total amount of uric acid in Uox^-/-^ rats excreted by urine and feces, as well as the total volume of urine, are equivalent or similar to those in humans (Table 2).

**Table 2:** Summary of Uox^-/-^ rats’ 24-h uric acid (UA) and the equivalence to that of men calculated by body surface area (men ± SD).

	n	SUA (μg/ml)	24h UA excreted(mg)	Urine (ml)
WT rats(/200g)	6	11.57 ± 3.63	4.29 ± 0.89	12.85 ± 2.78
Equivalent to men (60 kg)		-	205 ± 43	616 ± 133
Uox^-/-^ rats [30] (/200g)	53	64.4 ± 21.1	18.5 ± 12.3	32.41 ± 6.46
Equivalent to men (60 kg)		-	888 ± 598	1542 ± 310
Men (60 kg)		30–70	669 ± 173 (in urine only) [31]	~1500

### Uox^-/-^ rats are sensitive to purines and Xdh inhibitors

Wild-type rats’ SUA level is unsensitive to purines but adenine. Adenine is frequently used to elevate SUA in experimental studies [13], while other purines such as hypoxanthine and xanthine have rarely been used in wild-type rats although they can theoretically be converted to uric acid and raise the SUA level. Different from other purines, adenine can cause severe kidney damage with a mild but significant elevation of SUA [32]. Even when oxonate was jointly used, the elevation of SUA was still limited [32]. It has been reported that the elevation of SUA by adenine is actually the secondary effect to renal damage because adenine is easily converted to 2,8-dihydroxyadenine by Xdh [33, 34], a substance even less soluble than uric acid, and causes severe renal damage by obstructing glomeruli and tubules.

However, unlike wild-type rats, Uox^-/-^ rats are sensitive to purines and purine nucleosides other than adenine. Oral xanthine of 300 mg/kg for 5 days significantly elevated SUA levels from 60 μg/ml to 90 μg/ml, which was enough to diagnose hyperuricemia (above 70 μg/ml). As purine nucleosides, oral adenosine or inosine of 2.2 g/L by free drinking (about 660 mg/kg, equivalent of purine at 300 mg/kg) for 2 weeks can even elevate SUA level to 140 μg/ml or more. As expected, the SUA in Uox^-/-^ rats can be significantly lowered by Xdh inhibitors such as allopurinol.

In addition, Uox^-/-^ rats’ SUA level was elevated by alcohol and lowered by organic and inorganic salts of tris base, or by bicarbonate, suggesting that the intake of alkaline substances is helpful for lowering SUA. The amount of alkaline substances in a weakly alkaline mineral water is not sufficiently high to lower the SUA, although they can maintain pH of 9.3. Acetic acid did not lower SUA, but hydrochloric acid did. In addition, sodium acetate did not lower SUA, but the hydrochloride did. The results for acetate did not agree with the previous report [35] that also conflicted with an early study [36], and the cause could be the difference between the models. The results also suggested that the effect of SUA lowering is based on chloride ion rather than on sodium ion; they further suggested that uric acid could be inwardly transported through inorganic anion transporters [37], in addition to organic anion transporters [26, 38], which have been widely recognized.

### Uox^-/-^ rats’ renal function

As reported previously [17, 24], Uox^-/-^ rats are apparently healthy. Their serum creatinine and urea are at low levels in the normal ranges, although they are significantly higher than those in wild-type rats. However, the Uox^-/-^ rats’ renal function is vulnerable to factors other than can elevate SUA levels. If their SUA level is above 70 μg/ml, their serum creatinine and urea levels can exceed the reference ranges and were enough to diagnose renal injury. The results suggested that most of Uox^-/-^ rats’ renal reserve was lost.

### Kinetics of uric acid in Uox^-/-^ rats

Since SUA level in Uox^-/-^ rats is higher than that in wild-type rats, Uox^-/-^ rats are one of the best animals to explore the kinetics of uric acid. As mentioned above, purine metabolism in the rats is consistent with that in humans, and the results from the animal can be applied to humans to a large extent.

There are two sources of uric acid in the body. One is the main source synthesized by the intracellular enzymes, and the other one is synthesized by extracellular enzymes. Purines from the degradation of nuclei caused by cell turnover are a predominant endogenous source because the uric acid content in organs highly correlates with cell death [10]. Cell metabolism needs proteins that are translated from mRNA. Degradation of the mRNA and other RNA is another source of purines, but the proportion of uric acid from this source is likely minor. According to present understandings, the brain and the heart receive a large amount of blood and are regarded as highly metabolic organs. However, the uric acid content in the two organs was not as high as expected.

The extracellular fluids contain enzymes that participate in uric acid synthesis. For example, the activity of Xdh, the key enzyme to synthesize uric acid, can be usually detected in serum [17]. Xdh is a less selective enzyme that oxidizes hypoxanthine and xanthine to uric acid, and oxidizes adenine to 2,8-dihydroxyadenine [34]. The main task of the enzymes in extracellular fluids is to catalyze exogenous purines from food. It is not surprising: therefore, that oral purines do not generally increase uric acid levels in organs, but they do raise SUA levels.

Since the content of uric acid in organs is higher than that in serum, it can be considered that uric acid is mainly produced inside of cells rather than outside of cells, and then released into extracellular fluids, including blood. According to the present study, the main organs that synthesize uric acid are the liver, small intestine, and kidneys, because the key enzyme (Xdh) involved in uric acid synthesis is highly expressed in these sites. Interestingly, the content of uric acid in Uox^-/-^ rats’ organs (jejunum and ileum) was similar to that in wild-type rats and fasted wild-type rats [25], suggesting that the small intestine, in addition to the lung, colon, thymus, and brain, is a main organ for constitutive uric acid synthesis. Previous reports have supported the notion that the measures to increase the excretion of uric acid via the intestinal tract can also effectively lower SUA [25, 39]. Considering the kidney and liver are the organs whose uric acid level is most affected by factors that elevate SUA, they are inducible organs and the top two organs targeted at by the increased SUA.

Under normal circumstances, the kidney is the main excretion organ for uric acid. In Uox^-/-^ rats, more than 90% of uric acid is excreted by the kidneys, which obviously increases the burden on them. According to general understandings, the normal volume of final urine is about 1% of the primary urine because about 99% is reabsorbed by the kidneys. Based on the urine volume, it can be speculated that about 97% of the primary urine is reabsorbed in Uox^-/-^ rats, suggesting that the ability to reabsorb water is impaired. Since uric acid concentration in urine is about 9 times higher than that in blood, it can be calculated that more than 90% of uric acid in wild-type rats and more than 70% in Uox^-/-^ rats is reclaimed by the kidneys, suggesting that uric acid is not regarded as a thorough waste product by the body. This is consistent with literature reports [10], and also suggests that if SUA should be lowered by increasing renal excretion, the way to inhibit uric acid reabsorption is preferable, although the increase in uric acid secretion is still an effective way to lower SUA.

**Fig 8:**
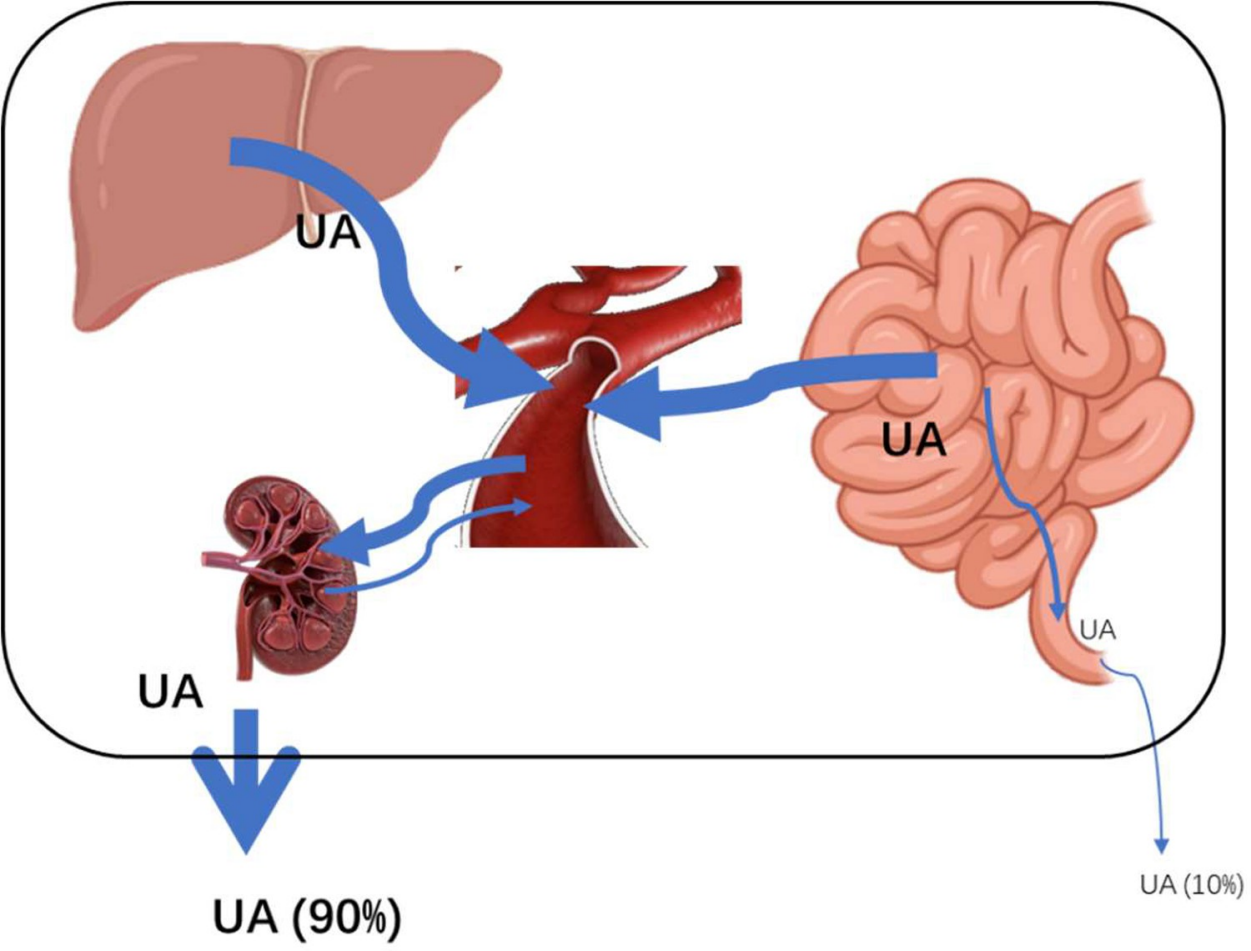
Kinetics of uric acid in Uox^-/-^ rats. Uric acid in the body is produced mainly by the liver, small intestine, and kidneys. Then, the uric acid is released into the blood, and excreted mostly through the kidneys.

The kinetics of uric acid circulation in Uox^-/-^ rats is summed up in Fig 8.

Interestingly, with the exception of the kidney, organs with uric acid content much higher than 70 μg/g rarely experience crystalline precipitation. Even in the kidneys, uric acid precipitation occurs in the extracellular fluid rather than in the intracellular fluid. The phenomenon suggested that there may be an unknown mechanism of uric acid solubilization in the intracellular fluid.

In conclusion, purine metabolism in Uox^-/-^ rats is consistent with that of humans, and Uox^-/-^ rats’ SUA level is sensitive to classical factors with expected effects. Uox^-/-^ rats are an alternative model animal for studying hyperuricemia and associated diseases.

## Supporting information

S1 Fig This the raw data for Fig 2. (XLSX)

S2 Fig Inosine adnosine.xlsx. This the raw data for Fig 3.
(XLSX)

S3 Fig This the raw data for Fig 4. (XLSX)

S4 Fig This the raw data for Fig 5. (XLSX)

S5 Fig This the raw data for Fig 6. (XLSX)

S6 Fig This the raw data for Fig 7. (XLSX)

S1 Table This the raw data for Table 1. (XLS)
